# VdAHA1 positively regulate pathogenicity in *Verticillium dahliae*

**DOI:** 10.3389/fmicb.2025.1535187

**Published:** 2025-05-26

**Authors:** Dongpo Li, Yuan Yuan, Jinglong Zhou, Zili Feng, Feng Wei, Heqin Zhu, Lihong Zhao, Hongjie Feng, Yalin Zhang

**Affiliations:** ^1^State Key Laboratory of Cotton Bio-breeding and Integrated Utilization, Institute of Cotton Research of Chinese Academy of Agricultural Sciences, Anyang, Henan, China; ^2^Western Agricultural Research Center of Chinese Academy of Agricultural Sciences, Changji, Xinjiang, China

**Keywords:** AHA1, *Verticillium dahliae*, microsclerotia, stress response, pathogenicity

## Abstract

AHA1 (activator of HSP90 ATPase) is a co-chaperone protein that mainly performs its function by interacting with the HSP90. The biological function of AHA1 has been widely reported in many species. In this study, we knocked out the *VdAHA1* gene of *V. dahliae* by homologous recombination method. The *VdAHA1* knockout mutants showed increased drug sensitivity to ergosterol synthesis pathway, significantly inhibiting ergosterol biosynthesis. The *VdAHA1* knockout mutant strain also showed decreased melanin in microsclerotia by reduced expression of microsclerotia and melanin related genes *Vaflm, Vayg1*, and *VdSCD*. The *VdAHA1* mutant showed decreased conidial production that were slightly damaged and showed more sensitivity to abiotic stresses such as temperature, SDS, CR, Sorbitol (SBT), NaCl, and KCl and decreased ATP contents. More importantly, the mutant was significantly less virulent to cotton than the wild type. This study identified the important functions of *VdAHA1* in the growth, stress resistance, and virulence.

## Introduction

*Verticillium dahliae* is a notorious plant pathogenic fungus. The mechanisms of virulence differentiation in *Verticillium dahliae* and its interaction with cotton remain unclear. Previous studies in our laboratory have shown that VdSti1, a core chaperone of heat shock proteins of *V. dahliae*, positively regulates virulence. At the same time, we investigated the function of VdAHA1 (activator of HSP90 ATPase), another core chaperone of HSP90. Heat shock protein 90 (HSP90) is a molecular chaperone that plays an important role in biological protein homeostasis (Mercier et al., 2023; Mondol et al., 2023; Siligardi et al., 2004). It is vital for the cell's response to stress and is a key player in the maintenance of cellular homeostasis (Jackson, 2013; Mielczarek-Lewandowska et al., 2020; Peng et al., 2022). The function of HSP90 in client protein processing is regulated by cochaperone molecules that modulate client protein activation in a specific manner, thereby influencing HSP90 ATPase activity and client protein recruitment (Biebl and Buchner, 2023; Rehn and Buchner, 2015). It has been reported that HSP90 influences pathogenicity. HSP90 is involved in the virulence of the opportunistic pathogen *Candida albicans* (Robbins and Cowen, 2023). Hsp90 proteins in *Aspergillus fumigatus* provide a mechanism to link temperature to signaling cascades that regulate morphogenesis, fungal development, and virulence (O'Meara and Cowen, 2014). In the opportunistic pathogen *Cryptococcus neoformans*, inhibition of HSP90 protein expressions makes it sensitive to temperature and antifungal azole drugs in turn reduce melanin synthesis and virulence (Fu et al., 2022). These studies all imply a potential role of HSP90 protein in fungal pathogenicity.

As a core co-chaperone of HSP90, AHA1 plays a pivotal role in modulating the chaperone cycle through its potent stimulation of HSP90's ATPase activity, which is achieved by regulating the allosteric dynamics of the HSP90 dimer (Oroz et al., 2019). AHA1 enhances the ATPase activity of HSP90 by specifically binding to the HSP90 dimer, thereby promoting ATP hydrolysis while simultaneously modulating both the catalytic efficiency and structural stability of the HSP90 complex (Mondol et al., 2023). In *Neurospora crassa* deletion of AHA1 resulted in greater sensitivity to azoles (Gu et al., 2016). Loss of AHA1 reduces the infectivity of *Leishmania donovani* in *ex-vivo* mouse macrophages (Bartsch et al., 2017). In *Saccharomyces cerevisiae*, AHA1 interacts with the HSP90 intermediate domain to regulate HSP90 ATPase activity (Lotz et al., 2003). These studies reveal the rich roles of AHA1 in various organisms (Xu et al., 2019).

Phytopathogenic fungi pose a major threat to global food security and cause significant losses to the global agricultural economy (Li P. et al., 2023; Wang et al., 2022; Wu et al., 2024). Soil-borne pathogens such as *Verticillium* and *Rhizoctonia* can cause serious plant diseases and are difficult to control (Ma et al., 2013; Peeters et al., 2013; Umer et al., 2023). *V. dahliae* is a notorious soil-borne pathogen that enters the host through its roots and multiplies in the ducts, causing Verticillium wilt (Carroll et al., 2018; Song et al., 2020; Zhang D. D. et al., 2022). Verticillium wilt poses a great challenge in host plants such as cotton and causes a great threat to the cotton industry with serious economic losses of hundreds of millions of dollars worldwide every year (Deketelaere et al., 2017; Zhang et al., 2023). To infect cotton, pathogens use multiple virulence mechanisms, such as the release of enzymes that degrade the cell wall, the activation of pathogenic genes, and use of protein effectors. In contrast, cotton plants have developed several defense mechanisms to combat *V. dahliae's* effects including the strengthening of the cell wall by the production of lignin and deposition of callose, the activation of reactive oxygen species, and the aggregation of hormones associated with defense (Umer et al., 2023). In fact, *V. dahliae* exhibits remarkable variability, co-evolution and complex pathogenic mechanisms (Luo et al., 2014). The specific mechanisms of interaction between plant pathogenic fungi and their hosts are not fully understood (Billah et al., 2021; Man et al., 2022; Zhu et al., 2023). Nonetheless, elucidating the pathogenic molecular mechanism of *V. dahliae* is expected to lay the foundation for solving Verticillium wilt threat to cotton.

AHA1 protein has been widely studied in various species such as human, animal, plant, and microbial, and exhibits various biological functions such as client protein folding and maturation, heat tolerance, adhesion, and virulence (LaPointe et al., 2020; Prodromou and Bjorklund, 2022). However, the function of AHA1 in *V. dahliae* has not been characterized. This study will provide valuable insights into the function of *VdAHA1* protein in plant pathogenic fungi. This study aims to investigate the function, pathway regulation, and virulence mechanism of *VdAHA1*, and to provide a theoretical basis to get rid of Verticillium wilt disease to ensure agricultural security.

## Materials and methods

### Growth conditions for plants and fungi

In this study, we used the Vd080 strain of *V. dahliae* (Wu et al., 2024). The fungi were cultured on potato dextrose agar (PDA) medium at 25°C in the dark. To collect fresh conidial fluid, the fungi were grown in liquid Czapek-Dox broth and shaken at 180 rpm for 6 days at 25°C. The known susceptible cotton variety, Jimian 11 (Mei et al., 2022; Miao et al., 2024), was used for the virulence test. The cotton plants were grown in a greenhouse at 25°C under a 16 h light/8 h dark photoperiod.

### Generation of knockout and complementation mutant strains

A homolog of AHA1 (termed AHA1) was found in the genome of *V. dahliae* strain VdLs.17. To obtain the knockout mutant strains, the 1.5 kb upstream and 1.5 kb downstream sequences of the target gene were amplified from the genomic DNA of *V. dahliae*. A hygromycin resistance cassette (HPH) was inserted into the B303 vector (Wen et al., 2023). The constructed vector was transformed into *Agrobacterium tumefacien* strain EHA105. The gene knockout mutant was constructed by infecting *V. dahliae* with positive *Agrobacterium tumefaciens* EHA105. The *A. tumefaciens* EHA105 suspension (OD_600_ = 0.6) was mixed with the *V. dahliae* conidial suspension (10^7^ CFU/mL) at a 1:1 (v/v) ratio for co-transformation (*Agrobacterium tumefaciens*-mediated transformation of filamentous fungi method, ATMT; Liu et al., 2023). A total of 300 μL of the mixture was evenly spread on the IM induction medium with hydrophilic membrane (supplemented with 400 μmol/L acetosyringone, AS) and incubated in the dark at 28°C for 48 h. Then the hydrophilic membrane was removed and transferred to PDA screening medium (Supplemented with cefalotin sodium 15 mg/L, carbenicillin 200 μg/mL, Hygromycin 35 μg/mL), and incubated at 25°C in the dark for 7 days. The single colonies were selected for PCR identification.

For complementation analysis, a 1.5 kb upstream regulatory fragment along with the complete coding sequence of the *VdAHA1* gene were amplified from the genomic DNA of *V. dahliae* and subsequently cloned into the pCAMBIA1302 vector. The resulting complementation vector was transformed into *A*. *tumefaciens* EHA105, and complementary strains were subsequently generated by ATMT method. To validate the successful generation of complementary mutants, RT-qPCR analysis was conducted to compare gene expression levels among the wild-type strain and the two complemented strains.

Protein multiple sequence alignment was performed using the online website ENDscript/ESPript. The specific primers were designed using the Primer-BLAST tool available on the NCBI website. The primers used in this experiment are listed in Supplementary Table S1.

### Determination of ergosterol content

Spore suspensions were inoculated into potato dextrose broth (PDB) medium and incubated at 25°C in the dark for 3 days. The PDB broth was filtered through eight layers of sterile gauze to collect the mycelia, which were subsequently dried using liquid nitrogen and ground into a fine powder. Five grams of the powdered mycelium were mixed with 25 mL of 10 mol/L NaOH solution and incubated in a 95°C water bath for 25 min. After incubation, the mixture was centrifuged at 12,000 rpm for 2 min to collect the solid precipitate. One gram of the precipitate was then resuspended in 10 mL of methanol and homogenized using a cell disruptor (Fisher Scientific Model 505, Thermo Fisher, Beijing, China) at 300 W (15 s on, 25 s off) for 25 min at room temperature. Following disruption, the supernatant was collected, and ergosterol concentrations were quantified by High Performance Liquid Chromatography (HPLC; Lv et al., 2023). Quantitative analysis was conducted using a Waters 2695 high-performance liquid chromatography (HPLC) system equipped with a Waters 2996 photodiode array detector. Chromatographic separation was achieved on a Waters C18 reverse-phase column (5 μm particle size, 4.6 mm internal diameter × 250 mm length) maintained at 30°C. The mobile phase consisted of 100% methanol (HPLC grade) with an isocratic elution at a flow rate of 1.0 mL/min. Ergosterol detection was performed at 282 nm with an injection volume of 20 μL (Dushkov et al., 2023; Fernandes et al., 2023). The experiment was repeated three times.

### Conidial yield, germination rate, ATP content and conidial morphology analysis

To evaluate conidial production across different strains, a 5 μL aliquot of conidial suspension (10^7^ CFU/mL) from the wild-type strain, knockout mutants, and complementation lines was inoculated onto the center of PDA plates. The inoculated plates were incubated at 25°C for 7 days. After incubation, an 8 mm diameter fungal plug was collected from the colony edge using a sterile cork borer. The fungal plug was transferred into 1 mL of sterile distilled water and vortexed thoroughly to release conidia. The conidial concentration was then determined using a hemocytometer (Li P. et al., 2023).

The concentration of the prepared conidial suspension was adjusted to 103 CFU/mL using sterile distilled water. For germination analysis, 50 μL aliquots of each conidial suspension were placed onto sterile glass slides and incubated in a humid chamber at 25°C for 16 h. Germination rates were determined by microscopic examination, with a conidium considered germinated when the germ tube length exceeded half the diameter of the conidium (Wang et al., 2023).

To investigate the ATP content of different strains, all strains were added to PDA medium and incubated at 25°C upside down in the dark for 7 days. Zero point one gram of mycelium was carefully excised from PDA cultures and homogenized in 1 mL of ice-cold distilled water using a tissue homogenizer maintained in an ice bath. The homogenate was then heat-treated at 100°C for 5 min, followed by centrifugation at 8,000 × g for 15 min at 4°C. The resulting supernatant was collected for subsequent ATP quantification. ATP content was determined through a creatine kinase-coupled enzymatic assay, where creatine kinase catalyzes the conversion of ATP and creatine to phosphocreatine (León et al., 1977). The reaction product was quantified using phosphomolybdic acid colorimetry, with absorbance measured at 700 nm after wavelength scanning to confirm the maximum absorption peak.

For morphological characterization of conidia, spores collected from the colony edge of PDA cultures were rapidly frozen in liquid nitrogen and subsequently coated with a thin gold layer under vacuum conditions. The prepared samples were then examined using scanning electron microscopy (SEM) to observe their ultrastructural features (Mei et al., 2022). The experiment was repeated three times.

### Abiotic stresses

To assess temperature sensitivity, conidia suspension (5 μL, 10^7^CFU/mL, prepared as previously described) was inoculated into the center of the PDA plate. The inoculated plates were incubated in complete darkness at five different temperatures (5°C, 15°C, 25°C, 30°C, and 35°C) for 14 days. Colony growth was monitored daily, and the radial growth diameter was measured at the end of the incubation period using a digital caliper. Three replicate plates were maintained for each temperature condition, and the experiment was repeated twice to ensure reproducibility.

For other abiotic stresses such as 0.9 mol/L KCl, 0.9 mol/L NaCl, 0.9 mol/L Sorbitol (SBT), 0.004% Sodium Dodecyl Sulfate (SDS), 0.02% Congo Red (CR), and 0.02% Calcofluor white (CFW) were inoculated to PDA medium (Zhang X. et al., 2022). Following the same experimental protocol as described for the temperature sensitivity analysis, fungal growth was monitored under identical conditions. The colony diameter was measured after a 14-day incubation period using a digital caliper, with three replicate plates for each condition and the experiment repeated twice to ensure data reliability.

To evaluate antifungal susceptibility, azole compounds targeting the ergosterol biosynthesis pathway, including ketoconazole (KTC, 1 mg/L), itraconazole (ITC, 5 mg/L), and fluconazole (FLU, 12.5 mg/L), were selected for sensitivity testing. The wild-type strain, knockout mutants, and complementation strains (10^7^ CFU/mL, prepared as described previously) were inoculated onto PDA plates supplemented with various concentrations of each azole compound. The inoculated plates were incubated at 25°C in complete darkness for 14 days, after which the colony diameters were measured using a digital caliper. Three replicate plates were maintained for each treatment condition, and the experiment was repeated twice to ensure reproducibility.

### Observation of microsclerotia

To investigate microsclerotia formation, 200 μL of freshly prepared conidial suspension was evenly spread onto basal minimal medium (BMM) plates containing sterile cellulose membranes. The plates were incubated at 25°C in complete darkness for 15 days. Microsclerotia were visualized using a stereomicroscope. The experiment was repeated three times.

### Pathogenicity assay

Fresh conidial suspensions were prepared from different *V. dahliae* strains and adjusted to a concentration of 1 × 10^6^ CFU/mL using a hemocytometer. Twenty-five-day-old cotton seedlings with intact root systems were carefully uprooted, and their roots were immersed in the conidial suspension for 10 min (Yu et al., 2019). The inoculated seedlings were then transplanted into nutrient-rich potting soil and cultivated in a cotton growth chamber for 21 days post-inoculation. Disease severity was assessed using a standardized disease index scale, and phenotypic symptoms were documented through digital photography. The plants were maintained under controlled environmental conditions with a 24-h photoperiod cycle: 16 h of light at 28°C followed by 8 h of darkness at 23°C, with relative humidity maintained at 60%−70%.

To determine the intensity of disease occurrence, symptoms in cotyledon and true leaves were graded from 0 to 4. Cotton stems collected 21 days post-inoculation (dpi) were longitudinally sectioned at the internode region using a sterile scalpel for vascular browning assessment. The degree of vascular discoloration was observed by stereoscopic microscope (Leica M205 FA, Germany) with digital image analysis.

For molecular analysis, stem samples from the same collection time point were immediately frozen in liquid nitrogen and homogenized to a fine powder using a pre-chilled mortar and pestle. Total genomic DNA was extracted using the Plant Genomic DNA Kit (DP305, Tiangen Biotech, Beijing, China) according to the manufacturer's protocol. RT-qPCR was used to quantify the colonization of *V. dahliae* in cotton stems. Primer information is provided in Supplementary Table S1.

### RT-qPCR analysis

For RNA extraction, all strains were cultured in potato dextrose broth (PDB) at 25°C with continuous shaking at 180 rpm for 6 days. The mycelia were collected by filtration through eight layers of sterile gauze and subsequently freeze-dried in liquid nitrogen. Total RNA was extracted using the FreeZol Reagent kit (Vazyme Biotech, Nanjing, China), followed by cDNA synthesis using the HiScript IV Reverse Transcriptase kit (Vazyme Biotech, Nanjing, China). The *V. dahliae*-specific β-tubulin gene (*Vd*β*t*) and cotton ubiquitin 7 gene (*GhUBQ7*) were used as reference genes. The thermal cycling protocol was conducted as follows: initial pre-denaturation at 95°C for 10 min, followed by 40 amplification cycles consisting of denaturation at 95°C for 15 s, primer annealing at 58°C for 30 s, and extension at 72°C for 30 s. The amplification was completed with a final extension step at 72°C for 10 min. To verify amplification specificity, melt curve analysis was performed following the amplification cycles. Relative gene expression levels across different samples were quantitatively analyzed using the 2^−ΔΔCT^ method, with each sample being analyzed in triplicate to ensure reproducibility (Livak and Schmittgen, 2001).

### RNA extraction and analysis of related gene expression

Total RNA of the corresponding strains was obtained using the method described above, after which cDNA libraries were constructed. The sequencing analysis was performed using the Illumina NovaSeq 6000 platform (Illumina, Shanghai, China), with a sequencing depth of 6 GB per sample. Differentially expressed genes (DEGs) were identified based on the following criteria: Expression change fold |log2FoldChange| > 1, significant *P*-value < 0.05. The relative expression levels of microsclerotia-related genes (*Vaflm, Vayg1*, and *VdSCD*); growth and development related genes (*VdNoxB, VdPls1, VdCrz1*); conidial growth and stress resistance related genes (*VdPLP, VdPf* , *VdERG2*); Virulence related genes (*VdKin2, VdCf2, hypothetical protein*) were quantified by RT-qPCR. Primer information is provided in Supplementary Table S1.

### Yeast two-hybrid screening and validation of interacting proteins

The CDS sequence of VdAHA1 was retrieved from the NCBI database, and specific primers were designed accordingly. Using the cDNA obtained in the previous step as a template, the CDS sequence of VdAHA1 was amplified by PCR. The target fragment was then ligated into the linearized PGBKT7 plasmid using a ClonExpress Ultra One Step Cloning Kit-C115 (Vazyme Biotech, Nanjing, China). The recombinant plasmid was verified by sequencing (Sangon Biotech, Shanghai, China) to ensure the correct insertion of the target fragment, resulting in the desired positive plasmid. Subsequently, the PGBKT7-VdAHA1 plasmid was used as bait to screen for interacting proteins from the *V. dahliae* yeast AD-cDNA library (preserved in our laboratory). The selected sequences were subjected to sequencing (Sangon Biotech, Shanghai, China), and the resulting data were analyzed by comparison with the NCBI database to identify the corresponding gene accession numbers. Specific primers were designed based on the obtained sequences. Using cDNA as the template, the target fragment was amplified and cloned into the linearized pGADT7 plasmid (ClonExpress Ultra One Step Cloning Kit-C115 Vazyme Biotech, Nanjing, China) and the recombinant plasmid was sequenced (Sangon Biotech, Shanghai, China) to ensure the correctness of the recombinant plasmid.

We performed a pairwise Yeast Two-Hybrid assay. The fusion plasmids of the two interacting proteins were transferred into yeast competent cells. The competent yeast cells were evenly spread on double dropout (DDO SD Leu Trp) medium. Subsequently, the yeast cultures showing normal growth on DDO plates were diluted to appropriate concentrations (10^−1^, 10^−2^, 10^−3^) and transferred to quadruple dropout (QDO SD His Leu Trp Ade) medium for further growth. After 1 week, X-α-gal staining was added dropwise and then observed. Positive control was used pGBKT7-53/pGADT7-RecT. Negative controls was used pGBKT7-Lam/pGADT7-RecT.

Specific primers were designed based on the corresponding CDS sequences, and recombinant plasmids VdAHA1-nLUC and VdHSP90-1-cLUC were constructed using the same method described above. The correctness of the plasmids was confirmed by sequencing (Sangon Biotech, Shanghai, China). Subsequently, the recombinant plasmids VdAHA1-nLUC and VdHSP90-1-cLUC were transformed into *Agrobacterium tumefaciens* AGL1 (Weidi Biological, Shanghai, China). Positive transformants were selected and inoculated into LB liquid medium, followed by overnight culture at 28°C with shaking at 180 rpm. When the OD_600_ of the bacterial suspension reached 0.6, fresh cultures were collected and mixed at a 1:1 ratio. The mixed bacterial solution was then injected into 4-week-old tobacco leaves. After injection, the tobacco plants were kept in the dark for 24 h and then transferred to normal growth conditions for an additional 24 h. Fluorescence signals in the tobacco leaves were detected using a low-light-cooled charge-coupled device (CCD) imaging system. Primer information is provided in Supplementary Table S1.

### Related information

Bioinformatics related website is provided in Supplementary Table S4; the related genes and corresponding information involved in the full text are shown in Supplementary Table S5.

## Results

### Identification of the *VdAHA1* gene in *V. dahliae*

**Figure 1:**
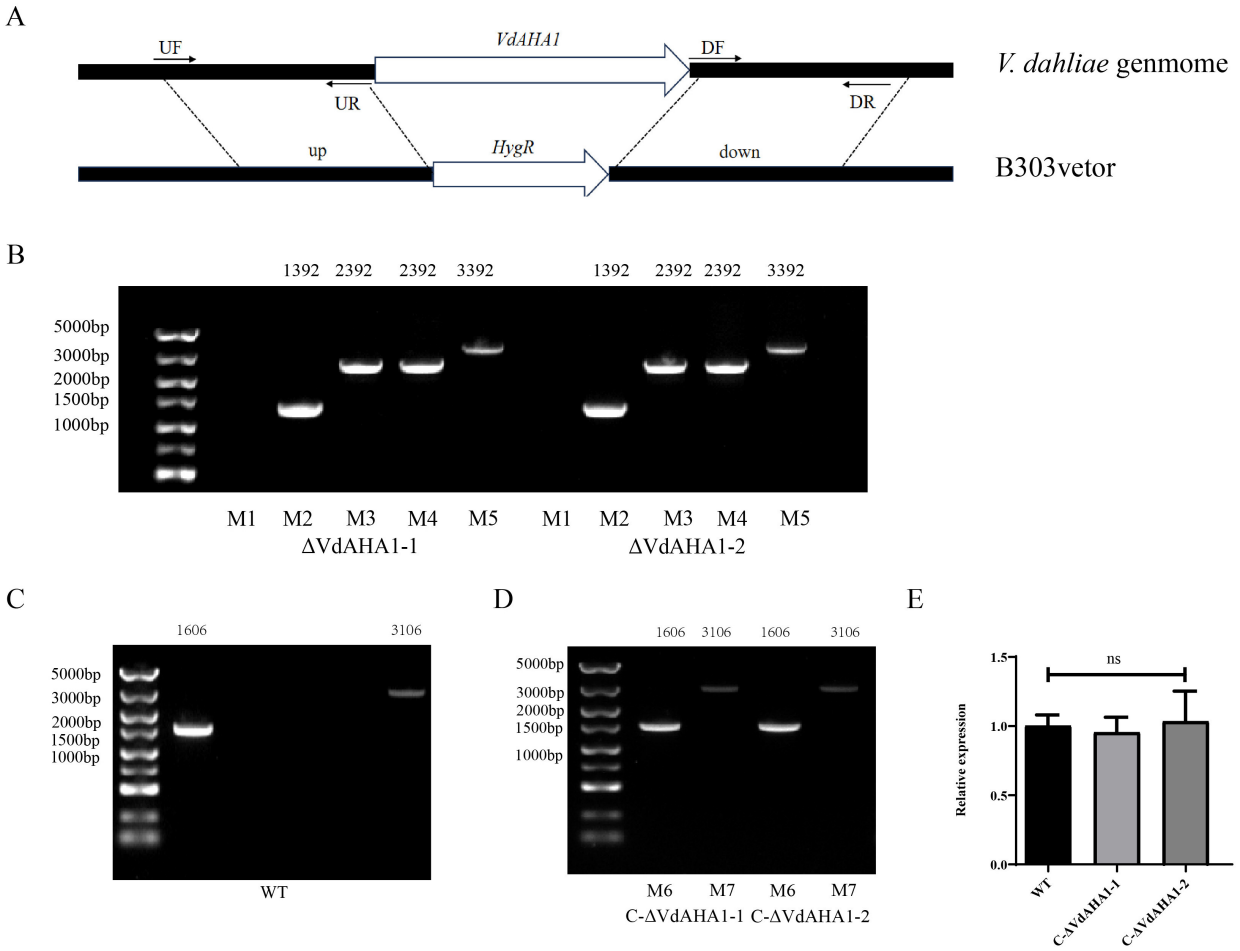
Knockout and complementary mutants were generated. **(A)** Mechanism of VdAHA1 gene knockout in *V. dahliae* VdAHA1. **(B–D)** Knockout strains and complementary strains were determined by PCR (M1: VdAHA1, M2: HPH, M3: UP1000 + VdAHA1, M4: DOWN1000 + VdAHA1, M5: UP1000 + DOWN1000 + VdAHA1, M6: VdAHA1, M7: UP1500 + VdAHA1). **(E)** Complementary strains were determined by RT-qPCR. The asterisks represent statistical differences performed by a *t*-test (ns, *p* < 0.01, *p < 0.05, **p < 0.01, and ***p < 0.001) in comparison with the wild type strains.

In our previous investigation, we identified VDAG_10067 as a significantly differentially expressed gene during the infection process of Vd080 in cotton roots (Wu et al., 2024). The NCBI database query showed that the gene was annotated as Hsp90 co-chaperone AHA1; therefore, we named the gene *VdAHA1* . The *VdAHA1* gene is 1,606 bp in length and encodes a 325-amino acid protein. Amino acid sequences of *VdAHA1* were compared with other *AHA1* homologs using the online website ENDscript/ESPript indicating that the AHA1 protein is highly conserved in all species (Supplementary Figure S1). To study the function of *VdAHA1* gene, *VdAHA1* gene knockout mutant strain and complement strain of *V. dahliae* were constructed by ATMT method (de Groot et al., 1998; Figure 1A). Two knockout mutants (ΔVdAHA1-1 and ΔVdAHA1-2) and two complementary strains (C-ΔVdAHA1-1 and C-ΔVdAHA1-2) were successfully generated. The results were confirmed by PCR and RT-qPCR (Figures 1B–E).

### The VdAHA1 knockout mutant shows higher azole sensitivity and lower ergosterol production than the wild type strain and the complementary strain

**Figure 2:**
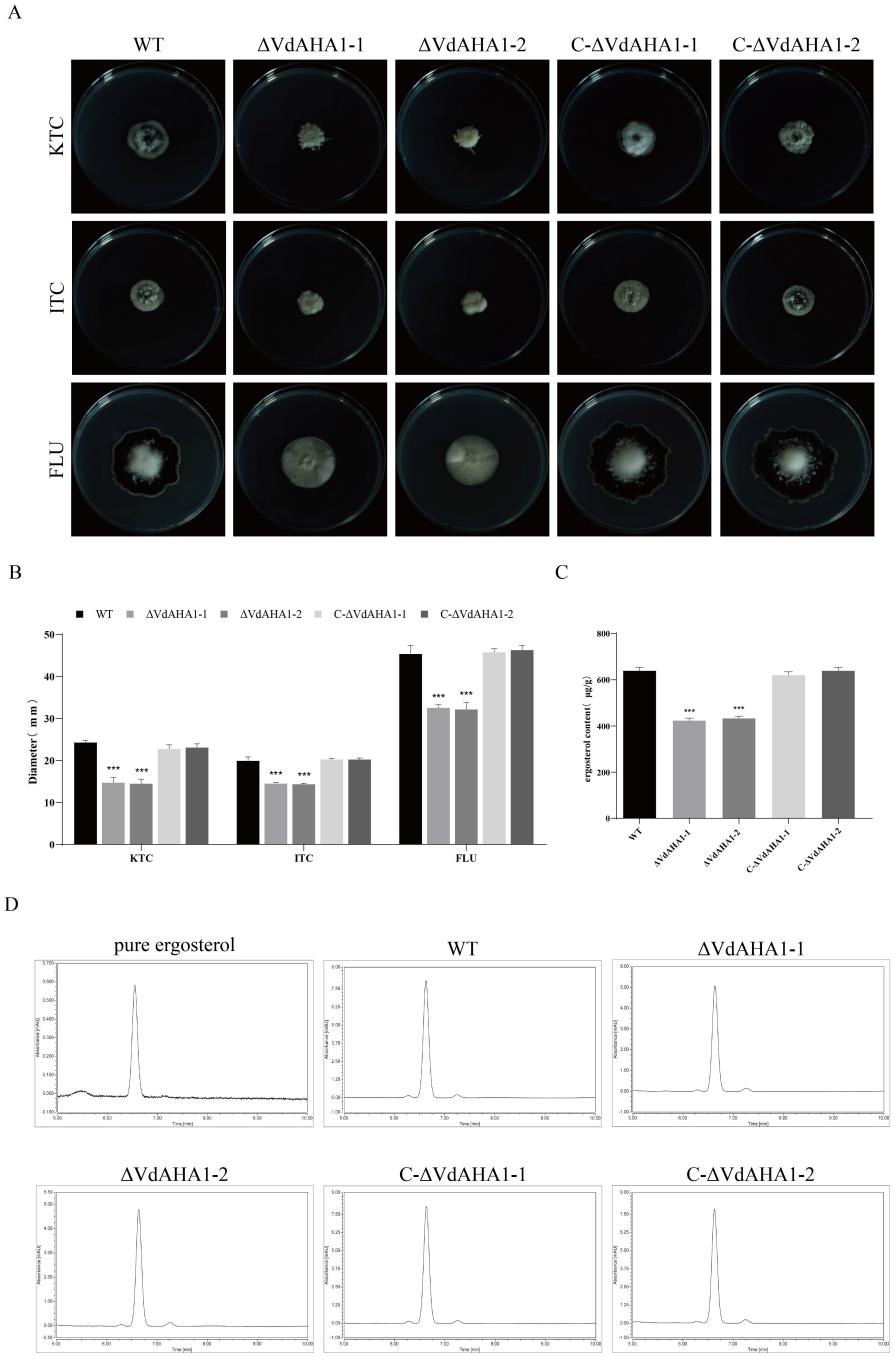
VdAHA1 deletion mutants showed different responses to different azole fungicides. Knockdown of VdAHA1 resulted in decreased ergosterol content. **(A)** The wild-type strain, the knockout mutant strain, and the complementary strain were incubated with 1 mg/L ketoconazole, 5 mg/L itraconazole, and 12.5 mg/L fluconazole at 25°C in the dark for 14 days. **(B)** All strains colony diameter. **(C)** Ergosterol content of all strains. The asterisks represent statistical differences performed by a *t*-test (*p < 0.05, **p < 0.01, and ***p < 0.001) in comparison with the wild type strains. **(D)** High performance liquid chromatography (HPLC) was used to determine the ergosterol content of wild-type, ΔVdAHA1 and C-ΔVdAHA1 strains. A commercial standard for ergosterol was used as a control.

It has been shown that AHA1 knockdown affects the sensitivity of fungi to azoles through the ergosterol synthesis pathway (Gu et al., 2016). All strains were tested for azole susceptibility and ergosterol content. The results showed that the VdAHA1 knockout mutant was more sensitive to azoles than the wild type strain and the complement strain (Figures 2A, B). These results suggest that the VdAHA1 plays a important role in the response of *V. dahliae* to anti-azole drugs. HPLC analysis of ergosterol extracted from mycelium of all strains showed that all strains had an ergosterol specific absorption peak with a retention time of 6.630 min. More importantly, the ergosterol content of the two knockout mutants was significantly lower than that of the wild type strain and complemented strain (Figures 2C, D). These results suggest a potentially critical role of VdAHA1 in the ergosterol synthesis pathway.

### VdAHA1 responds to abiotic stresses

**Figure 3:**
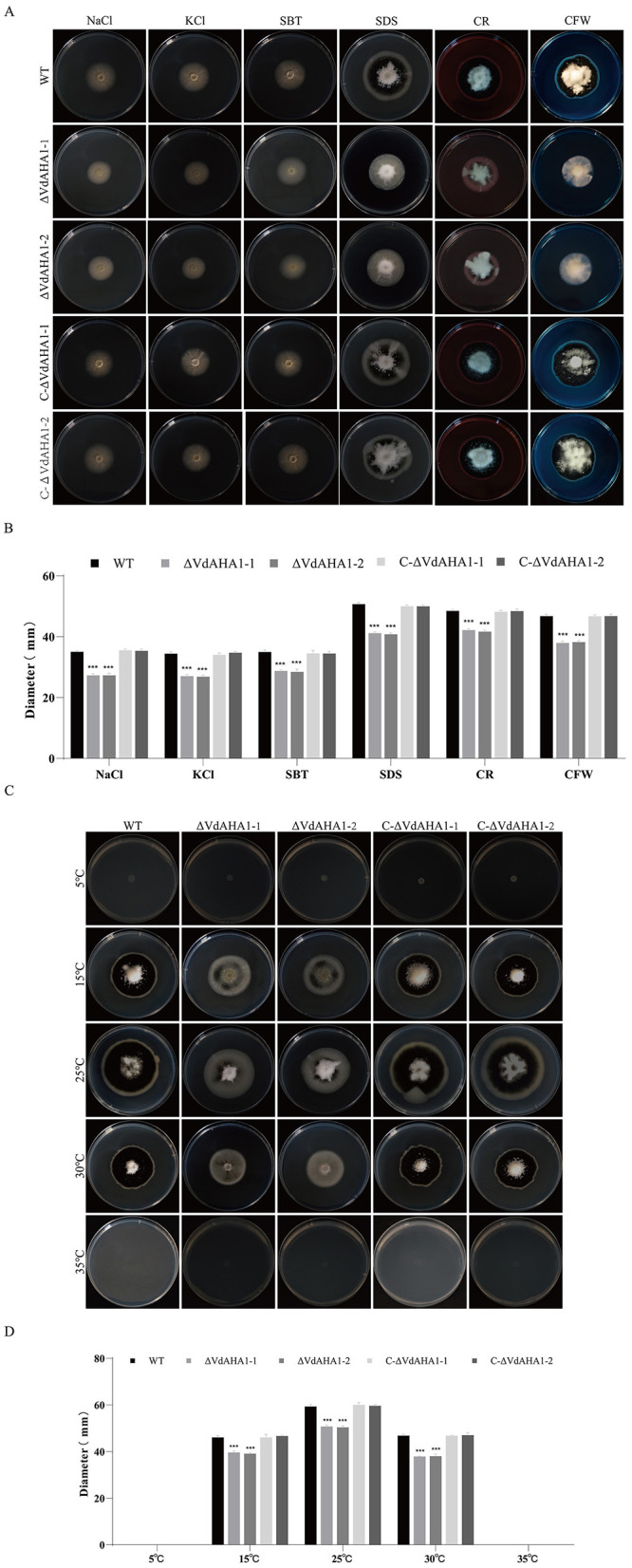
VdAHA1 responded to cell wall, membrane, osmotic, and temperature stresses in *V. dahliae*. **(A)** The wild-type strain, knockout mutant strain, and complement strain were cultured in the dark on PDA plates containing 0.9 mol/L KCl, 0.9 mol/L NaCl, 0.9 mol/L SBT, 0.004% SDS, 0.02% CR, and 0.02% CFW for 14 days. **(C)** Colony morphology of all strains cultured on PDA plates at 5°C, 15°C, 25°C, 30°C, 35°C in dark for 14 days. **(B, D)** Colony diameters of different strains. Values represent means ± standard deviation of three replicates. The asterisks represent statistical differences performed by a *t*-test in comparison with the wild type strains (*p < 0.05, **p < 0.01, and ***p < 0.001).

To evaluate the abiotic stress responses of the knockout mutant strains, we assessed their tolerance to osmotic stress (KCl, NaCl, SBT) and wall/membrane perturbants (CR, SDS, CFW). VdAHA1 knockout mutants showed compromised growth under osmotic and cell wall/membrane stressors (Figures 3A, B). To investigate the role of VdAHA1 in temperature stress, we measured the colony diameter of *VdAHA1* knockout mutants at different temperatures. The results showed that the growth of the knockout mutant was significantly inhibited at 15°C, 25°C, and 30°C compared to the wild-type and complementary strains. Meanwhile, all strains were unable to grow at 5°C and 35°C (Figures 3C, D).

### VdAHA1 is vital for normal conidial function

**Figure 4:**
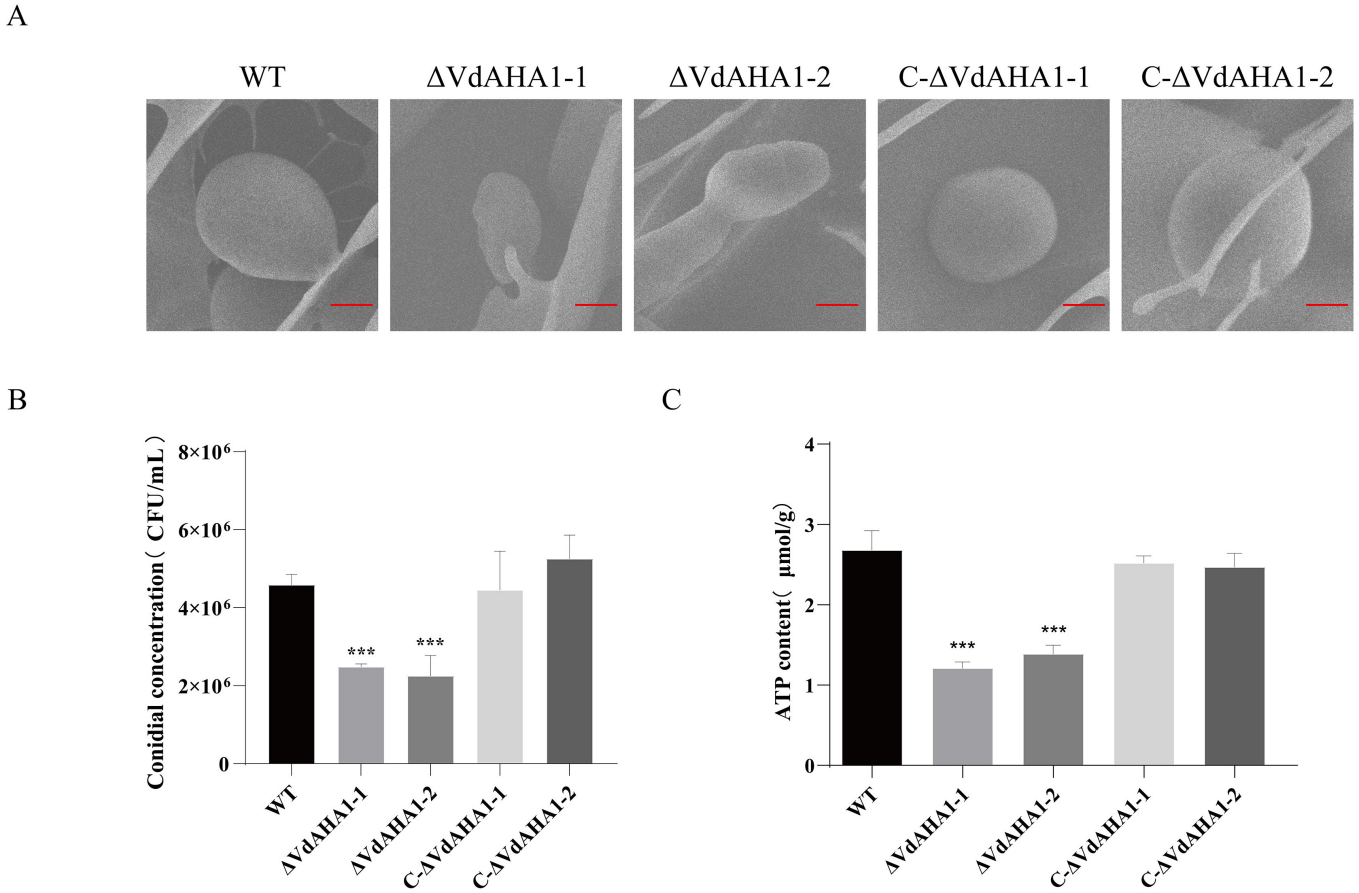
Deletion of VdAHA1 resulted in impaired conidial growth. **(A)** Under electron scanning microscopy, the mutant strain showed slight damage to the conidial morphology. Bar = 20 μm. **(B, C)** Conidial production and ATP content were significantly reduced in the knockout mutants. Values represent means ± standard deviation of three replicates. The asterisks represent statistical differences performed by a *t*-test (*p < 0.05, **p < 0.01, and ***p < 0.001) in comparison with the wild type strains.

Studies have shown that AHA1 interacts with HSP90 to participate in a variety of complex physiological and biochemical functions by affecting the ATPase activity of HSP90 protein. The spore yield and germination rate of all strains were investigated. The ATP content of each strain was also determined. At the same time, the conidial morphology of all strains was observed. The results showed that the knockout mutants' conidial yield and ATP content were significantly decreased (Figures 4A–C). Meanwhile, the germination rate of conidia also decreased significantly (Supplementary Figures S2A, B). It is worth mentioning that the knockout mutants showed a slight impairment in conidial morphology (Figure 4A).

### The VdAHA1 is positively involved in the production of microsclerotia and melanin in *V. dahliae*

**Figure 5:**
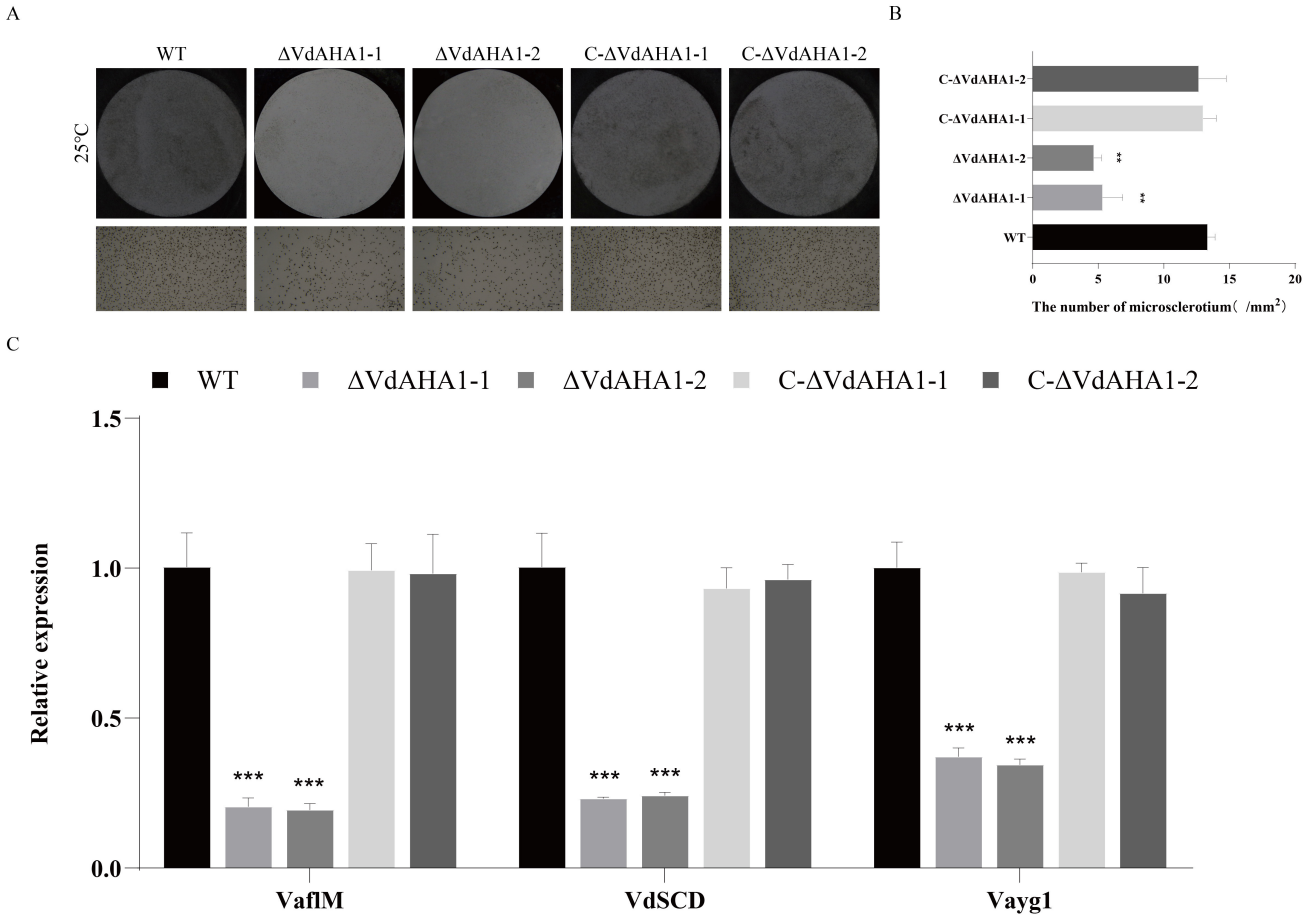
Deletion of VdAHA1 in *V. dahliae* resulted in decreased microsclerotia. **(A)** Microsclerotia growth of all strains after 15 days of incubation in the dark on BMM solid medium containing nitrocellulose membrane. **(B)** Number of mm^2^ microsclerotia in all strains. **(C)** RT-qPCR was used to detect the expression of related genes *VaflM, VdSCD*, and *Vayg1*. Values represent means ± standard deviation of three replicates. The asterisks represent statistical differences performed by a *t*-test in comparison with the wild type strains (*p < 0.05, **p < 0.01, and ***p < 0.001).

To investigate the relationship between VdAHA1 and melanin and micronucleus formation in *V. dahliae*, we measured melanin and micronucleus formation on solid basal medium (BMM) with cellulose film for 15 days (Li H. et al., 2023). Melanin and micronucleus production were significantly reduced in *VdAHA1* knockout mutants (Figures 5A, B). To further understand the function of VdAHA1 in the microsclerotium and melanin production of *V. dahliae*, a panel of related genes was analyzed. The results showed that the expression of related factors was significantly reduced in the knockout mutant strain. All results indicating that VdAHA1 positively regulates melanin and micronucleus formation, and is involved in the expression of related genes (Figure 5C).

### VdAHA1 positively regulates the pathogenicity of *V. dahliae*

**Figure 6:**
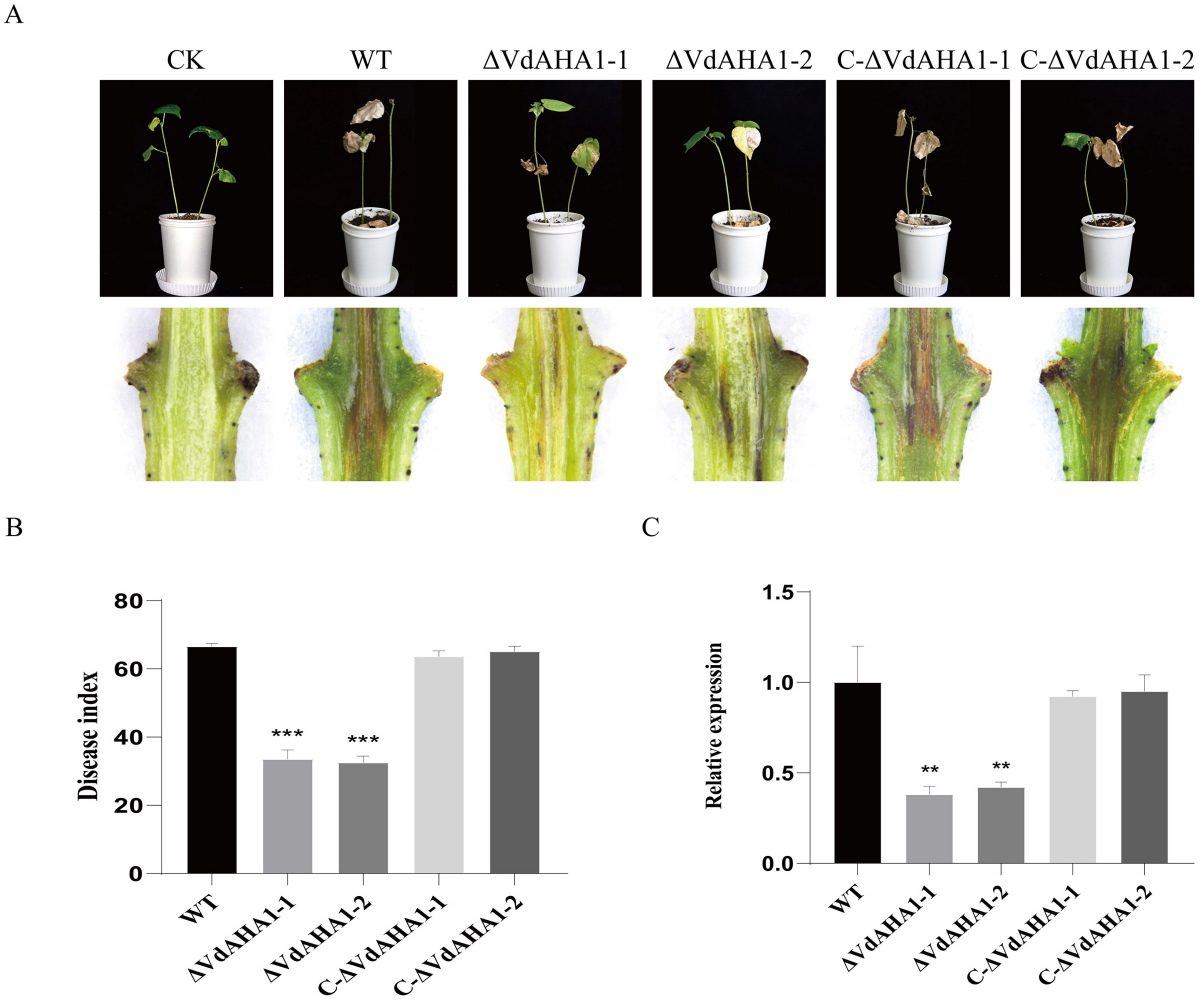
VdAHA1 positively regulates the pathogenicity of *V. dahliae*. **(A)** Symptoms of cotton inoculated with different strains. Cotton stems are browning. Photographs were taken 21 days after fungal inoculation. **(B)** Disease index of cotton inoculated with different strains. **(C)** Different strains were infected 21 days. The biomass of the knockout mutant strain decreased significantly in the cotton stem. *Vd*β*t* was used as the detection gene, and *GhUBQ7* of upland cotton was used as the endogenous control gene. Values represent means ± standard deviation of three replicates. The asterisks represent statistical differences performed by a *t*-test in comparison with the wild type strains (*p* < 0.01, *p < 0.05, **p < 0.01, and ***p < 0.001).

To further investigate whether VdAHA1 contributes to the virulence of *V. dahliae* on cotton, we performed pathogenicity assays with all strains. The spore solution of all strains was inoculated into cotton. Pure water was used as a control. After 21 days incubation, marked symptoms of verticillium and necrosis of leaves were observed in both the wild and complemented strains (Figure 6A). In contrast, the disease index of plants infected with ΔVdAHA1-1 and ΔVdAHA1-2 mutants decreased significantly (Figure 6B). Cotton stems inoculated with the *VdAHA1* knockout mutants showed less browning than the wild-type (Figure 6A). To further investigate the effect of VdAHA1 on the pathogenicity of *V. dahliae*, the biomass of all strains was determined by qRT-PCR. The results showed that the biomass of the two knockout mutants was significantly reduced compared with the wild type strain and the complemented strain, respectively (Figure 6C). These results suggest that *VdAHA1* positively regulates the pathogenicity of *V. dahliae*.

### RNA-seq analysis of VdAHA1 gene knockout mutants

**Figure 7:**
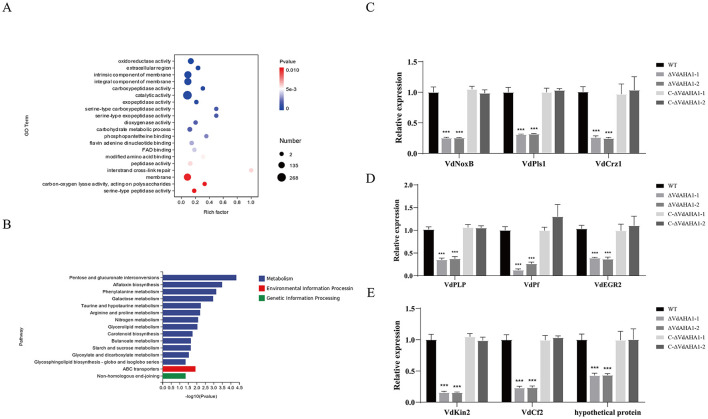
VdAHA1 is involved in a variety of related pathways. **(A)** Gene ontology enrichment of DEGs from ΔVdAHA1 vs. WT. Gene ratio is the number of DEGs divided by the total number of genes associated with a specific pathway. **(B)** Knockdown mutants downregulated KEGG enrichment of DEGs compared to wild-type strains. **(C)** The genes related to growth and development of *V. dahliae* were down-regulated. **(D)** The genes related to stress resistance of *V. dahliae* were down-regulated. **(E)** Virulence-related genes of *V. dahliae* were down-regulated. The asterisks represent statistical differences performed by a *t*-test in comparison with the wild type strains (*p* < 0.01, *p < 0.05, **p < 0.01, and ***p < 0.001).

To better understand the potential function of VdAHA1 in *V. dahliae*, we analyzed the differentially expressed genes (DEGs) in ΔVdAHA1s using RNA sequencing (RNA-Seq). RNA-seq results identified 891 differentially expressed genes in the ΔVdAHA1s, including 366 up-regulated genes and 525 down-regulated genes (Supplementary Figure S3A) elucidated that the changes in gene expression is caused by *VdAHA1* knockdown in *V. dahliae*. Go terms and KEGG enrichment analyses were performed to better understand the functions of down-regulated genes. The top 20 significantly enriched Go term pathways, including oxidoreductase, carboxypeptidase, catalytic, exopeptidase activity, extracellular regions, membrane intrinsic components, carbohydrate metabolic processes, and inter-chain cross-link repair pathways (Figure 7A) are composed of 99, 8, 268, 12, 178, 177, 12, and 11 differentially expressed genes (DEGs), respectively. The figure showed the top 15 KEGG pathways were significantly enriched in pentose and glucuronate interconversion, aflatoxin biosynthesis, phenylalanine metabolism, and galactose metabolism, containing 11, 3, 6, and 6 DEGs, respectively (Figure 7B). We randomly selected 10 downregulated candidate genes from the enrichment pathways and analyzed the expression levels of the wild-type and knockout mutants. All 10 candidate genes were downregulated in the knockout mutants compared to the wild-type (Supplementary Figure S3B). The results of RT-qPCR and RNA-Seq were consistent (Supplementary Figure S3C). To further understand the function of VdAHA1 in cotton interaction, we screened the previously reported genes in DEGs. The RT-qPCR results showed that the expression levels of these genes were significantly decreased, which was consistent with the above results (Figures 7C–E). These results indicate that deletion of the *VdAHA1* gene in *V. dahliae* significantly alters the expression levels of multiple functional genes involved in diverse signaling pathways. Furthermore, these findings provide new insights into the functional roles of *VdAHA1* in *V. dahliae*.

### VdAHA1 interacts with VdHSP90-1

**Figure 8:**
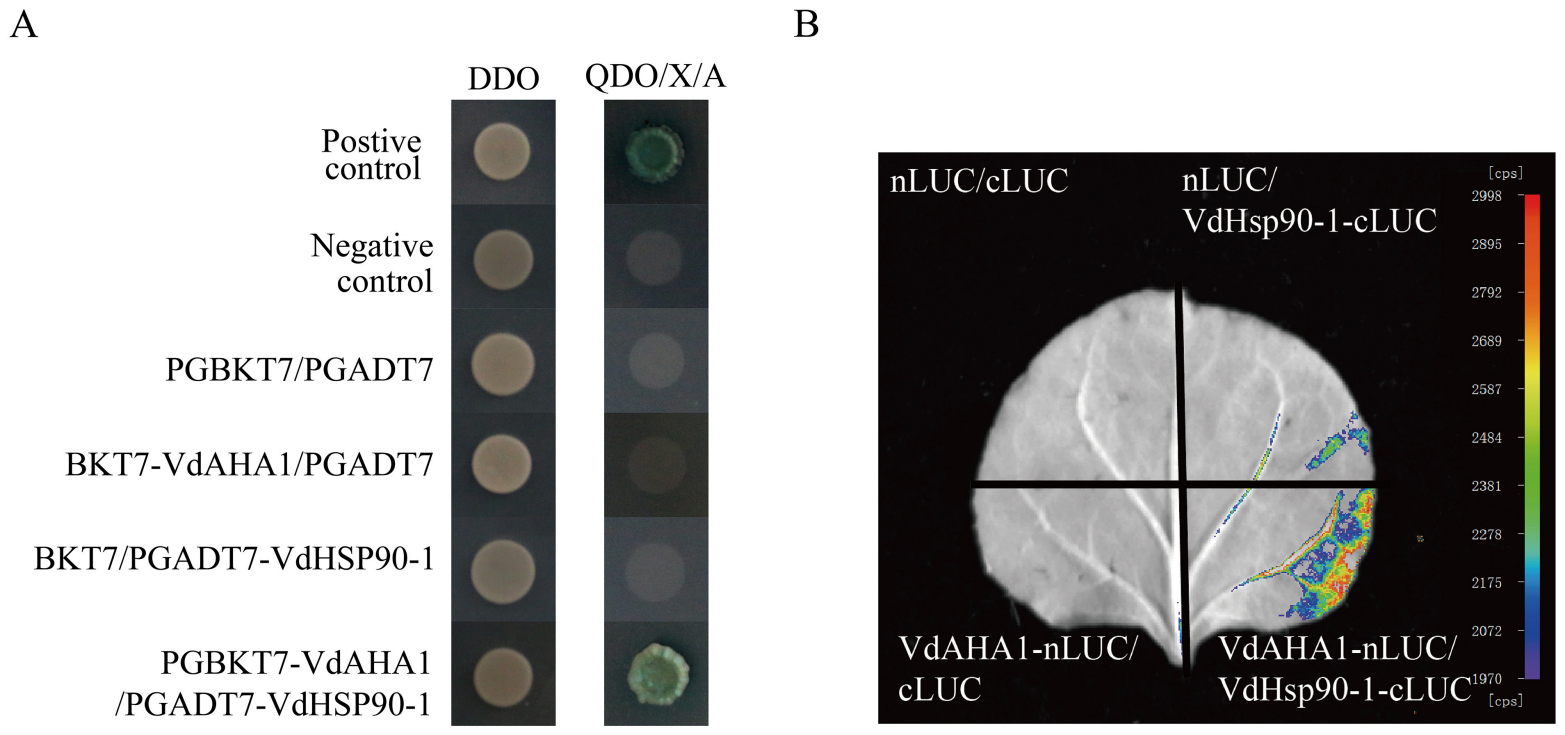
VdAHA1 interacts with VdHSP90-1. **(A)** Y2H assays of the interactions of VdAHA1 with VdHSP90-1. Positive control: pGBKT7-53/pGADT7-RecT. Negative controls: pGBKT7-Lam/pGADT7-RecT. **(B)** The interaction of VdAHA1 with VdHSP90-1 was detected by LCI.

To further elucidate the functional roles of VdAHA1 in *V. dahliae*, we performed yeast two-hybrid screening to identify its interacting proteins. The coding sequence (CDS) of VdAHA1 was cloned into the pGBKT7 bait vector for yeast two-hybrid screening. Subsequent screening of a *Verticillium dahliae* cDNA library identified VdHSP90-1 as a specific interaction partner of VdAHA1 (Figure 8A, Supplementary Figure S4). To further validate these findings, we performed luciferase complementation assays (LCI), which confirmed the interaction between VdAHA1 and VdHSP90-1 (Figure 8B). In addition, we performed a joint analysis of the screening library results with the RNA-Seq results. The results showed that most of the DEGs in the results of screening cDNA libraries were hypothetical proteins (Supplementary Tables S2, S3).

## Discussion

Our previous study identified VdAHA1 as a differentially expressed gene during *V. dahliae* (strain Vd080) infection of cotton roots. Bioinformatics analysis revealed that VdAHA1 is annotated in the NCBI database as an Hsp90 co-chaperone AHA1, suggesting its potential role through chaperone-mediated protein folding.

Ergosterol is essential for maintaining fungal membrane properties, requiring sterols without a C-4 methyl group for proper function. Azole drugs target the cytochrome P-450-dependent 14α-demethylase, inhibiting ergosterol biosynthesis and leading to the accumulation of 14α-methylated sterol precursors (Alcazar-Fuoli and Mellado, 2013; Da Silva Ferreira et al., 2005). Studies have shown that HSP90, under the regulation of comolecular chaperone AHA1, can stabilize key enzymes in the ergosterol biosynthesis pathway, such as CYP51/Erg11, and enhance azole resistance in fungi by inhibiting the proteasome-mediated degradation pathway (Cowen and Lindquist, 2005). In *Neurospora crassa*, knockout of the *AHA1* gene resulted in a notable accumulation of toxic sterols, especially 14α-methyl-3,6-diol, as demonstrated by HPLC. This evidence not only underscores the crucial role of AHA1 in modulating cellular membrane homeostasis, but also elucidates its pivotal function in conferring antifungal drug resistance. At the transcriptional level, *AHA1* positively regulates the response to ketoconazole stress through the key genes erg11 and erg6 in the ergosterol biosynthesis pathway (Yu et al., 2007). In the present study, deletion of the *VdAHA1* gene in *V. dahliae* resulted in a significant decrease in ergosterol content and increased sensitivity to azole antifungal agents. These findings suggest that AHA1 represents a promising target for the development of novel antifungal agents.

The HSP90-cochaperone system is a master regulator of essential cellular processes, including proteostasis, cell cycle control, hormone signaling, and apoptotic regulation (Hoter et al., 2018). The experimental results that VdAHA1 positively regulates abiotic stress are compatible with the function of HSP90. In particular, there are more insights on temperature stress. The evolutionarily conserved Hsp90 chaperone system functions as a thermal sensor regulating fungal morphogenesis, development, and virulence (O'Meara and Cowen, 2014). Recent studies have revealed that in *Candida albicans*, a major human fungal pathogen, Hsp90 regulates both drug resistance and virulence. *Candida albicans* exhibits temperature-dependent morphological changes that modulate its virulence, though the precise molecular regulators of this process remain to be fully elucidated (Shapiro and Cowen, 2012). In *Ruditapes philippinarum*, it has been shown that temperature stress is a key regulator in the HSP90 and HSP70 biological regulatory network (Jahan et al., 2023). While current evidence does not establish a direct causal relationship between the VdAHA1-VdHSP90-1 interaction and the observed abiotic stress responses, we consider this regulatory mechanism a plausible hypothesis worthy of further investigation.

AHA1 protein is involved in a variety of cellular functions (Biebl and Buchner, 2023) and showed its positive implications for agricultural security and the economy. AHA1 acts as a core co-chaperone of HSP90, which is able to stimulate ATP hydrolysis of HSP90 (Mondol et al., 2023). The interaction of HSP90 with the AHA1 co-chaperone (Lepvrier et al., 2015) implies that HSP90 is required for AHA1 function. In this study, the *VdAHA1* knockout mutant exhibited a substantial reduction in intracellular ATP levels, suggesting a functional association between VdAHA1 and HSP90 in energy metabolism. This interaction was subsequently validated through direct experimental evidence demonstrating the interaction between VdAHA1 and VdHSP90-1. We speculate that VdAHA1 contributes to the above results by affecting the function of VdHSP90-1.

Verticillium wilt of cotton, caused by *V. dahliae*, is a devastating vascular disease. The pathogen produces highly resilient microsclerotia (dormant structures) that can persist in soil for extended periods, contributing significantly to the challenges in disease management and control (Li et al., 2024). Many genes have been shown to regulate microsclerotia production, including VdKss1, VdSti1, VdHog1, VDH1, VdPbs2, VdZFP1, and VdZFP2. VdKss1 has been reported to regulate the production of microsclerotium and virulence of *V. dahliae*. On the contrary, VdSti1, which also serves as a core chaperone molecule of heat shock proteins, negatively regulates the formation of microsclerotia and melanin (Li et al., 2024; Wu et al., 2024). Deletion of VdHog1, a mitogen-activated protein kinase (MAPK), led to a significant reduction in both micronuclei formation and melanin production. In the ΔVdHog1 strain, the expression levels of genes associated with melanin biosynthesis and the hydrophobic protein gene VDH1, which plays a critical role in the early stages of micronucleus formation, were markedly downregulated. The conserved upstream component of VdHog1, VdPbs2, serves as a critical regulator of micronucleus formation in *V. dahliae*. It plays a pivotal role in positively regulating the development of microsclerotia and the biosynthesis of melanin, highlighting its essential function in these key biological processes. VdZFP1 and VdZFP2 act as positive regulators of VdCmr1, facilitating melanin deposition during micronucleus development (Li H. et al., 2023; Tian et al., 2016; Wang et al., 2016). In our study, we found that VdAHA1 positively regulated the formation of melanin and microsclerotia. Phenotypic analyses revealed that VdAHA1 deletion leads to: (i) reduced conidial production and germination rate, (ii) decreased ATP content, and (iii) mild morphological alterations in conidia. However, whether this process is related to the function of HSP90 protein and whether it is directly related to the virulence of the *V. dahliae* are still unclear.

The virulence mechanisms of *V. dahliae* and its interaction with cotton plants constitute a highly complex biological system that remains incompletely understood to date. In one study, 34 candidate effector proteins in the secretory proteome of *V. dahliae* were analyzed and identified. The glycoside hydrolase family 11 protein Vd424Y was found to be significantly up-regulated at the early stage of *V. dahliae* infection in cotton. This protein is located in the nucleus and its deletion impairs fungal virulence (Liu et al., 2021). During cotton infection, VdEG1 and VdEG3 function as PAMPs and virulence factors, respectively (Gui et al., 2017). VdEPG1 is an important virulence factor of *V. dahliae* (Liu et al., 2023). However, HSP90 is an essential chaperone protein that protects the integrity of the proteome, accounts for 2% of cellular proteins, and is required for exosome release (Lauwers et al., 2018). Knockout of HSP70 and HSP90 cochaperone Sti1 in *V. dahliae* resulted in decreased pathogenicity (Wu et al., 2024). According to our results, VdAHA1 positively regulates the virulence of *V. dahliae*. Specifically, we selected and validated several downregulated genes identified in the knockout mutants through RT-qPCR analysis. These included: (i) developmental regulators (VdNoxB, VdPls1, VdCrz1), (ii) stress-responsive genes (VdPLP, VdPf, VdERG2), and (iii) virulence-associated factors (VdKin2, VdCf2, and hypothetical proteins). The RT-qPCR results were consistent with our RNA-seq data.

Through comparative analysis between potential interacting proteins identified from cDNA library screening and DEGs, we observed that most candidate interactors were hypothetical proteins. We analyzed that, on the one hand, the function of the assumed protein has not been deeply studied, and on the other hand, the screening library results are highly likely to be false positive. Future investigations should focus on validating these protein-protein interactions and elucidating the biological functions of the identified interacting proteins.

## Conclusion

Our study provides valuable insights into the function and mechanism of *VdAHA1* in *V. dahliae*. Elucidating its roles in pathogenesis and growth will provide insight into this important fungal pathogen. These results extend our knowledge of the AHA1 family proteins of fungal pathogens and open new avenues for the control of Verticillium wilt in cotton. In the future, targeted new pesticides can be developed based on the role of VdAHA1 in ergosterol synthesis. Future studies should explore the underlying molecular mechanism and potential intervention targets of *VdAHA1* based on its function to better control Verticillium wilt.

## Data Availability

The original contributions presented in the study are included in the article/Supplementary material, further inquiries can be directed to the corresponding authors.

## Supplementary material

Supplementary Figure S1 The amino acid sequence alignment of the AHA1 structural domain (genotype of AHA1 in *Verticillium dahliae, Colletotrichum chlorophyti, Fusarium avenaceum, Staphylotrichum tortipilum*, and *Schizosaccharomyces pombe*). Numbers represent the position of the amino acid (aa) residues.

Supplementary Figure S2 The conidial germination rate of the knockout mutant was significantly lower than that of the wild type and complementation mutant. **(A)** Conidia germinated at 25°C for 8 and 16 h. bar = 100 μm. **(B)** Germination rate of all strains. Values represent means ± standard deviation of three replicates. The asterisks represent statistical differences performed by a *t*-test in comparison with the wild type strains (^*^*p* < 0.05, ^**^*p* < 0.01, and ^***^*p* < 0.001).

Supplementary Figure S3 RNA-Seq results. **(A)** In EDGs, 366 genes were up-regulated and 525 genes were down-regulated. **(B)** Heatmap of 10 downregulated genes in ΔVdAHA1. **(C)** RT-qPCR was used to detect the expression of related genes. Values represent means ± standard deviation of three replicates.

Supplementary Figure S4 Yeast two-hybrid assays showed that VdAHA1 interacts with VdHSP90-1.

Supplementary Table S1 Primers used in this study.

Supplementary Table S2 Gene number and annotation.

Supplementary Table S3 Gene number and annotation.

Supplementary Table S4 Bioinformatics related website.

Supplementary Table S5 Gene number and annotation.

## References

[B1] Alcazar-Fuoli L. Mellado E. (2013). Ergosterol biosynthesis in *Aspergillus fumigatus*: its relevance as an antifungal target and role in antifungal drug resistance. Front. Microbiol. 3:439. 10.3389/fmicb.2012.00439 23335918 PMC3541703

[B2] Bartsch K. Hombach-Barrigah A. Clos J. (2017). Hsp90 inhibitors radicicol and geldanamycin have opposing effects on *Leishmania* AHA1-dependent proliferation. Cell Stress Chaperones 22, 729–742. 10.1007/s12192-017-0800-2 28455612 PMC5573691

[B3] Biebl M. M. Buchner J. (2023). p23 and AHA1 : distinct functions promote client maturation. Subcell Biochem. 101, 159–187. 10.1007/978-3-031-14740-1_6 36520307

[B4] Billah M. Li F. Yang Z. (2021). Regulatory network of cotton genes in response to salt, drought and wilt diseases (*Verticillium* and *Fusarium*): progress and perspective. Front. Plant Sci. 12:759245. 10.3389/fpls.2021.759245 34912357 PMC8666531

[B5] Carroll C. L. Carter C. A. Goodhue R. E. Lawell C. C. L. Subbarao K. V. (2018). A review of control options and externalities for Verticillium wilts. Phytopathology 108, 160–171. 10.1094/PHYTO-03-17-0083-RVW 28703041

[B6] Cowen L. E. Lindquist S. (2005). Hsp90 potentiates the rapid evolution of new traits: drug resistance in diverse fungi. Science 309, 2185–2189. 10.1126/science.1118370 16195452

[B7] Da Silva Ferreira M. E. Colombo A. L. Paulsen I. Ren Q. Wortman J. Huang J.. (2005). The ergosterol biosynthesis pathway, transporter genes, and azole resistance in *Aspergillus fumigatus*. Med. Mycol. 43, S313–S319. 10.1080/13693780400029114 16110826

[B8] de Groot M. J. Bundock P. Hooykaas P. J. Beijersbergen A. G. (1998). *Agrobacterium tumefaciens*-mediated transformation of filamentous fungi. Nat. Biotechnol. 16, 839–842. 10.1038/nbt0998-839 9743116

[B9] Deketelaere S. Tyvaert L. França S. C. Höfte M. (2017). Desirable traits of a good biocontrol agent against Verticillium wilt. Front. Microbiol. 8:1186. 10.3389/fmicb.2017.01186 28729855 PMC5498563

[B10] Dushkov A. Vosáhlová Z. Tzintzarov A. Kalíková K. Krížek T. Ugrinova I. (2023). Analysis of the ibotenic acid, muscimol, and ergosterol content of an amanita muscaria hydroalcoholic extract with an evaluation of its cytotoxic effect against a panel of lung cell lines *in vitro*. Molecules 28:6824. 10.3390/molecules28196824 37836667 PMC10574166

[B11] Fernandes C. Sousa-Baptista J. Lenha-Silva A. F. Calheiros D. Correia E. Figueirinha A.. (2023). Azorean black tea (*Camellia sinensis*) antidermatophytic and fungicidal properties. Molecules 28:7775. 10.3390/molecules28237775 38067505 PMC10707949

[B12] Fu C. Beattie S. R. Jezewski A. J. Robbins N. Whitesell L. Krysan D. J.. (2022). Genetic analysis of Hsp90 function in *Cryptococcus neoformans* highlights key roles in stress tolerance and virulence. Genetics 220:iyab164. 10.1093/genetics/iyab164 34849848 PMC8733452

[B13] Gu X. Xue W. Yin Y. Liu H. Li S. Sun X. (2016). The Hsp90 co-chaperones Sti1, AHA1, and P23 regulate adaptive responses to antifungal azoles. Front. Microbiol. 7:1571. 10.3389/fmicb.2016.01571 27761133 PMC5050212

[B14] Gui Y. J. Chen J. Y. Zhang D. D. Li N. Y. Li T. G. Zhang W. Q.. (2017). *Verticillium dahliae* manipulates plant immunity by glycoside hydrolase 12 proteins in conjunction with carbohydrate-binding module 1. Environ. Microbiol. 19, 1914–1932. 10.1111/1462-2920.13695 28205292

[B15] Hoter A. El-Sabban M. E. Naim H. Y. (2018). The HSP90 family: structure, regulation, function, and implications in health and disease. Int. J. Mol. Sci. 19:2560. 10.3390/ijms19092560 30158430 PMC6164434

[B16] Jackson S. E. (2013). Hsp90: structure and function. Top. Curr. Chem. 328, 155–240. 10.1007/128_2012_356 22955504

[B17] Jahan K. Nie H. Yan X. (2023). Revealing the potential regulatory relationship between HSP70, HSP90 and HSF genes under temperature stress. Fish Shellfish Immunol. 134:108607. 10.1016/j.fsi.2023.108607 36758653

[B18] LaPointe P. Mercier R. Wolmarans A. (2020). Aha-type co-chaperones: the alpha or the omega of the Hsp90 ATPase cycle? Biol. Chem. 401, 423–434. 10.1515/hsz-2019-0341 31782942

[B19] Lauwers E. Wang Y. C. Gallardo R. Van der Kant R. Michiels E. Swerts J.. (2018). Hsp90 mediates membrane deformation and exosome release. Mol. Cell 71, 689–702.e9. 10.1016/j.molcel.2018.07.016 30193096

[B20] León L. P. Sansur M. Snyder L. R. Horvath C. (1977). Continuous-flow analysis for glucose, triglycerides, and ATP with immobilized enzymes in tubular form. Clin. Chem. 23, 1556–1562. 10.1093/clinchem/23.9.1556 19165

[B21] Lepvrier E. Moullintraffort L. Nigen M. Goude R. Allegro D. Barbier P.. (2015). Hsp90 oligomers interacting with the AHA1 cochaperone: an outlook for the Hsp90 chaperone machineries. Anal. Chem. 87, 7043–7051. 10.1021/acs.analchem.5b00051 26076190

[B22] Li H. Sheng R. C. Zhang C. N. Wang L. C. Li M. Wang Y. H.. (2023). Two zinc finger proteins, VdZFP1 and VdZFP2, interact with VdCmr1 to promote melanized microsclerotia development and stress tolerance in *Verticillium dahliae*. BMC Biol. 21:237. 10.1186/s12915-023-01697-w 37904147 PMC10617112

[B23] Li P. Tedersoo L. Crowther T. W. Wang B. Shi U. Juang L.. (2023). Global diversity and biogeography of potential phytopathogenic fungi in a changing world. Nat. Commun. 14:6482. 10.1038/s41467-023-42142-4 37838711 PMC10576792

[B24] Li W. Li S. Tang C. Klosterman S. J. Wang Y. (2024). Kss1 of *Verticillium dahliae* regulates virulence, microsclerotia formation, and nitrogen metabolism. Microbiol. Res. 281:127608. 10.1016/j.micres.2024.127608 38241914

[B25] Liu L. Wang Z. Li J. Wang Y. Yuan J. Zhan J.. (2021). *Verticillium dahliae* secreted protein Vd424Y is required for full virulence, targets the nucleus of plant cells, and induces cell death. Mol. Plant Pathol. 22, 1109–1120. 10.1111/mpp.13100 34233072 PMC8358993

[B26] Liu S. Liu R. Lv J. Feng Z. Wei F. Zhao L.. (2023). The glycoside hydrolase 28 member VdEPG1 is a virulence factor of *Verticillium dahliae* and interacts with the jasmonic acid pathway-related gene GhOPR9. Mol. Plant Pathol. 24, 1238–1255. 10.1111/mpp.13366 37401912 PMC10502839

[B27] Livak K. J. Schmittgen T. D. (2001). Analysis of relative gene expression data using real-time quantitative PCR and the 2(-Delta Delta C(T)) method. Methods 25, 402–408. 10.1006/meth.2001.1262 11846609

[B28] Lotz G. P. Lin H. Harst A. Obermann W. M. (2003). AHA1 binds to the middle domain of Hsp90, contributes to client protein activation, and stimulates the ATPase activity of the molecular chaperone. J. Biol. Chem. 278, 17228–17235. 10.1074/jbc.M212761200 12604615

[B29] Luo X. Xie C. Dong J. Yang X. Sui A. (2014). Interactions between *Verticillium dahliae* and its host: vegetative growth, pathogenicity, plant immunity. Appl. Microbiol. Biotechnol. 98, 6921–6932. 10.1007/s00253-014-5863-8 24928658

[B30] Lv J. Liu S. Zhang X. Zhao L. Zhang T. Zhang Z.. (2023). VdERG2 was involved in ergosterol biosynthesis, nutritional differentiation and virulence of *Verticillium dahliae*. Curr. Genet. 69, 25–40. 10.1007/s00294-022-01257-9 36416932

[B31] Ma L. J. Geiser D. M. Proctor R. H. Rooney A. P. O'Donnell K. Trail F.. (2013). Fusarium pathogenomics. Annu. Rev. Microbiol. 67, 399–416. 10.1146/annurev-micro-092412-155650 24024636

[B32] Man M. Zhu Y. Liu L. Luo L. Han X. Qiu L.. (2022). Defense mechanisms of cotton fusarium and verticillium wilt and comparison of pathogenic response in cotton and humans. Int. J. Mol. Sci. 23:12217. 10.3390/ijms232012217 36293072 PMC9602609

[B33] Mei J. Wu Y. Niu Q. Miao M. Zhang D. Zhao Y.. (2022). Integrative analysis of expression profiles of mRNA and microRNA provides insights of cotton response to *Verticillium dahliae*. Int. J. Mol. Sci. 23:4702. 10.3390/ijms23094702 35563093 PMC9099760

[B34] Mercier R. Yama D. LaPointe P. Johnson J. L. (2023). Hsp90 mutants with distinct defects provide novel insights into cochaperone regulation of the folding cycle. PLoS Genet. 19:e1010772. 10.1371/journal.pgen.1010772 37228112 PMC10246838

[B35] Miao F. Chen W. Zhao Y. Zhao P. Sang X. Lu J.. (2024). The RING-type E3 ubiquitin ligase gene GhDIRP1 negatively regulates *Verticillium dahliae* resistance in cotton (*Gossypium hirsutum*). Plants 13:2047. 10.3390/plants13152047 39124165 PMC11314081

[B36] Mielczarek-Lewandowska A. Hartman M. L. Czyz M. (2020). Inhibitors of HSP90 in melanoma. Apoptosis 25, 12–28. 10.1007/s10495-019-01577-1 31659567 PMC6965345

[B37] Mondol T. Silbermann L. M. Schimpf J. Vollmar L. Hermann B. Tych K. K.. (2023). AHA1 regulates Hsp90′s conformation and function in a stoichiometry-dependent way. Biophys. J. 122, 3458–3468. 10.1016/j.bpj.2023.07.020 37515325 PMC10502475

[B38] O'Meara T. R. Cowen L. E. (2014). Hsp90-dependent regulatory circuitry controlling temperature-dependent fungal development and virulence. Cell Microbiol. 16, 473–481. 10.1111/cmi.12266 24438186

[B39] Oroz J. Blair L. J. Zweckstetter M. (2019). Dynamic AHA1 co-chaperone binding to human Hsp90. Protein Sci. 28, 1545–1551. 10.1002/pro.3678 31299134 PMC6699087

[B40] Peeters N. Guidot A. Vailleau F. Valls M. (2013). Ralstonia solanacearum, a widespread bacterial plant pathogen in the post-genomic era. Mol. Plant Pathol. 14, 651–662. 10.1111/mpp.12038 23718203 PMC6638647

[B41] Peng C. Zhao F. Li H. Li L. Yang Y. Liu F. (2022). HSP90 mediates the connection of multiple programmed cell death in diseases. Cell Death Dis. 13:929. 10.1038/s41419-022-05373-9 36335088 PMC9637177

[B42] Prodromou C. Bjorklund D. M. (2022). Advances towards understanding the mechanism of action of the Hsp90 complex. Biomolecules 12:600. 10.3390/biom12050600 35625528 PMC9138868

[B43] Rehn A. B. Buchner J. (2015). p23 and AHA1. Subcell Biochem. 78, 113–131. 10.1007/978-3-319-11731-7_6 25487019

[B44] Robbins N. Cowen L. E. (2023). Roles of Hsp90 in *Candida albicans* morphogenesis and virulence. Curr. Opin. Microbiol. 75:102351. 10.1016/j.mib.2023.102351 37399670 PMC11016340

[B45] Shapiro R. S. Cowen L. E. (2012). Uncovering cellular circuitry controlling temperature-dependent fungal morphogenesis. Virulence 3, 400–404. 10.4161/viru.20979 22722238 PMC3478242

[B46] Siligardi G. Hu B. Panaretou B. Piper P. W. Pearl L. H. Prodromou C. (2004). Co-chaperone regulation of conformational switching in the Hsp90 ATPase cycle. J. Biol. Chem. 279, 51989–51998. 10.1074/jbc.M410562200 15466438

[B47] Song R. Li J. Xie C. Jian W. Yang X. (2020). An overview of the molecular genetics of plant resistance to the Verticillium wilt pathogen *Verticillium dahliae*. Int. J. Mol. Sci. 21:1120. 10.3390/ijms21031120 32046212 PMC7037454

[B48] Tian L. Wang Y. Yu J. Xiong D. Zhao H. Tian C. (2016). The mitogen-activated protein kinase kinase VdPbs2 of *Verticillium dahliae* regulates microsclerotia formation, stress response, and plant infection. Front. Microbiol. 7:1532. 10.3389/fmicb.2016.01532 27729908 PMC5037172

[B49] Umer M. J. Zheng J. Yang M. Batool R. Abro A. A. Hou Y.. (2023). Insights to Gossypium defense response against *Verticillium dahliae*: the cotton cancer. Funct. Integr. Genom. 23:142. 10.1007/s10142-023-01065-5 37121989

[B50] Wang D. Wen S. Zhao Z. Long Y. Fan R. (2023). Hypothetical protein VDAG_07742 is required for *Verticillium dahliae* pathogenicity in potato. Int. J. Mol. Sci. 24:3630. 10.3390/ijms24043630 36835042 PMC9965449

[B51] Wang Y. Pruitt R. N. Nürnberger T. Wang Y. (2022). Evasion of plant immunity by microbial pathogens. Nat. Rev. Microbiol. 20, 449–464. 10.1038/s41579-022-00710-3 35296800

[B52] Wang Y. Tian L. Xiong D. Klosterman S. J. Xiao S. Tian C. (2016). The mitogen-activated protein kinase gene, VdHog1, regulates osmotic stress response, microsclerotia formation and virulence in *Verticillium dahliae*. Fungal Genet. Biol. 88, 13–23. 10.1016/j.fgb.2016.01.011 26812120

[B53] Wen Y. Zhou J. Feng H. Sun W. Zhang Y. Zhao L.. (2023). VdGAL4 modulates microsclerotium formation, conidial morphology, and germination to promote virulence in *Verticillium dahliae*. Microbiol. Spectr. 11:e0351522. 10.1128/spectrum.03515-22 36475739 PMC9927093

[B54] Wu Y. Zhou J. Wei F. Zhang Y. Zhao L. Feng Z.. (2024). The role of VdSti1 in *Verticillium dahliae*: insights into pathogenicity and stress responses. Front. Microbiol. 15:1377713. 10.3389/fmicb.2024.1377713 38638896 PMC11024458

[B55] Xu W. Beebe K. Chavez J. D. Boysen M. Lu Y. Zuehlke A. D.. (2019). Hsp90 middle domain phosphorylation initiates a complex conformational program to recruit the ATPase-stimulating cochaperone AHA1. Nat. Commun. 10:2574. 10.1038/s41467-019-10463-y 31189925 PMC6561935

[B56] Yu J. Li T. Tian L. Tang C. Klosterman S. J. Tian C.. (2019). Two *Verticillium dahliae* MAPKKKs, VdSsk2 and VdSte11, have distinct roles in pathogenicity, microsclerotial formation, and stress adaptation. mSphere 4:e00426-19. 10.1128/mSphere.00426-19 31292234 PMC6620378

[B57] Yu L. Zhang W. Wang L. Yang J. Liu T. Peng J.. (2007). Transcriptional profiles of the response to ketoconazole and amphotericin B in *Trichophyton rubrum*. Antimicrob. Agents Chemother. 51, 144–153. 10.1128/AAC.00755-06 17060531 PMC1797652

[B58] Zhang D. D. Dai X. F. Klosterman S. J. Subbarao K. V. Chen J. Y. (2022). The secretome of *Verticillium dahliae* in collusion with plant defence responses modulates Verticillium wilt symptoms. Biol. Rev. Camb. Philos. Soc. 97, 1810–1822. 10.1111/brv.12863 35478378 PMC9542920

[B59] Zhang X. Zhao L. Liu S. Zhou J. Wu Y. Feng Z.. (2022). Identification and functional analysis of a novel hydrophobic protein VdHP1 from *Verticillium dahliae*. Microbiol. Spectr. 10:e0247821. 10.1128/spectrum.02478-21 35377232 PMC9045179

[B60] Zhang Y. Zhang Y. Ge X. Yuan Y. Jin Y. Wang Y.. (2023). Genome-wide association analysis reveals a novel pathway mediated by a dual-TIR domain protein for pathogen resistance in cotton. Genome Biol. 24:111. 10.1186/s13059-023-02950-9 37165460 PMC10170703

[B61] Zhu Y. Zhao M. Li T. Wang L. Liao C. Liu D. (2023). Interactions between *Verticillium dahliae* and cotton: pathogenic mechanism and cotton resistance mechanism to Verticillium wilt. Front. Plant Sci. 14:1174281. 10.3389/978-2-8325-1321-7 37152175 PMC10161258

