# Supplementary figures and images for: VdAHA1 positively regulate pathogenicity in Verticillium dahliae
Source: Front Microbiol. 2025 May 26;16:1535187. doi: 10.3389/fmicb.2025.1535187 (PMC12146375; doi:10.3389/fmicb.2025.1535187)

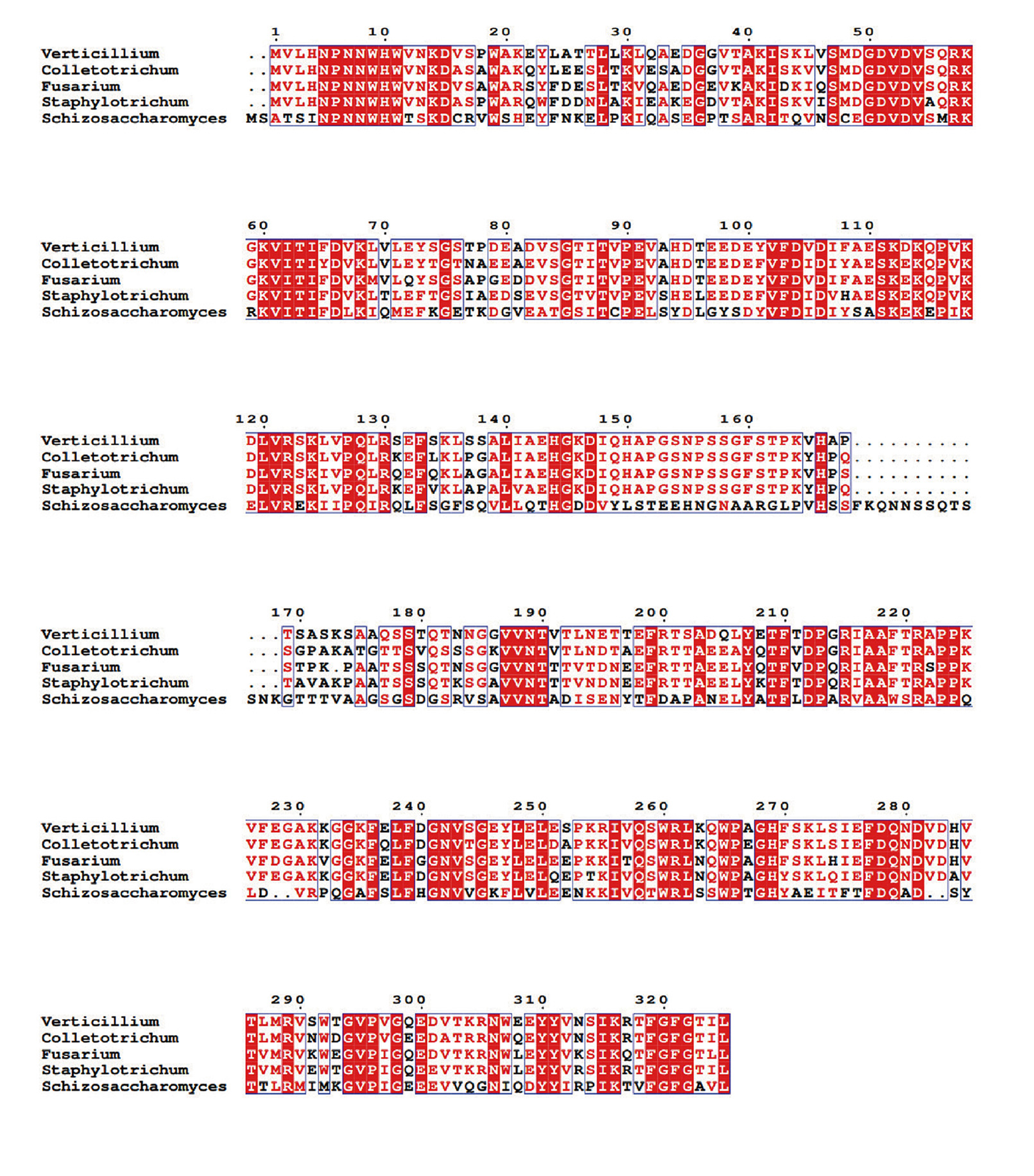

Supplement: Supplementary Figure S1 — The amino acid sequence alignment of the AHA1 structural domain (genotype of AHA1 in Verticillium dahliae, Colletotrichum chlorophyti, Fusarium avenaceum, Staphylotrichum tortipilum, and Schizosaccharomyces pombe). Numbers represent the position of the amino acid (aa) residues. [file Image_1.jpeg]

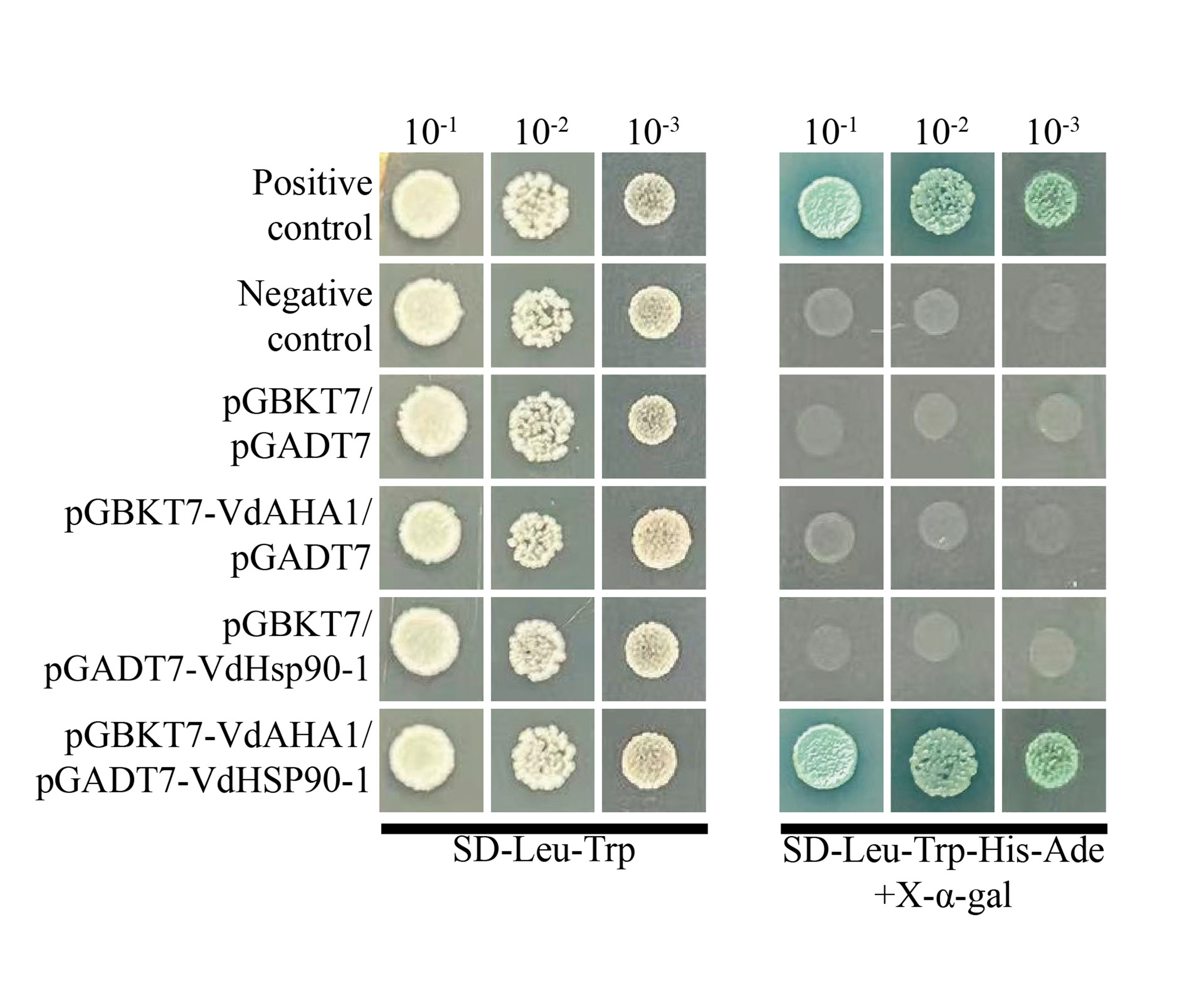

Supplement: Supplementary Figure S4 — Yeast two-hybrid assays showed that VdAHA1 interacts with VdHSP90-1. [file Image_4.jpeg]
