# Supplementary material for: VdAHA1 positively regulate pathogenicity in Verticillium dahliae
Source: Front Microbiol. 2025 May 26;16:1535187. doi: 10.3389/fmicb.2025.1535187 (PMC12146375; doi:10.3389/fmicb.2025.1535187)
Supplement: Supplementary Table S1 — Primers used in this study. [file Table_1.docx]

**Table S1**

| Primers name | Primer sequence (5’-3’) | restriction site | use |
| --- | --- | --- | --- |
| VdAHA1-F | ACCGAAAACACGTCCACAGGTAAG |  | PCR |
| VdAHA1-R | GGGCATCAACATGTAGATGCAAG |  | PCR |
| Hyg-F | GACGTTAACTGATATTGAAG |  | PCR |
| Hyg-R | CTATTCCTTTGCCCTCGGACG |  | PCR |
| B303-VdAHA1-UP-F | GGACCGGACGGGGCGGTACC  GACACCAACCAAGACCCGGAC | *Kpn*I | deletion of VdAHA1 |
| B303-VdAHA1-UP-R | CTTCAATATCAGTTAACGTC  GTCGTTATCTCATAGCTAAGGACAC |  | deletion of VdAHA1 |
| B303-VdAHA1-Hyg-F | GTGTCCTTAGCTATGAGATAACGAC  GACGTTAACTGATATTGAAG |  | deletion of VdAHA1 |
| B303-VdAHA1-Hyg-R | GACGAGTTTTGAGCGACAAAGAT  CTATTCCTTTGCCCTCGGACG |  | deletion of VdAHA1 |
| B303-VdAHA1-DOWN-F | CGTCCGAGGGCAAAGGAATAG  ATCTTTGTCGCTCAAAACTCGTC |  | deletion of VdAHA1 |
| B303-VdAHA1-DOWN-R | TTAATTAAGACCCGGGACTA  GCGAAGATGGGCATAATTAGAAC | *Pac*I | deletion of VdAHA1 |
| BIA1302-VdAHA1-C-F | ACGGCCAGTGCCAAGCTT  GACACCAACCAAGACCCGGAC | *Bamh*I | Complem  entation of VdAHA1 |
| BIA1302-VdAHA1-C-R | CGTTCTCGGAGGAGGCCAT GGGCATCAACATGTAGATGCAAG |  | Complem  entation of VdAHA1 |
| BIA1302-VdAHA1-RFP-F | CTTGCATCTACATGTTGATGCCC  ATGGCCTCCTCCGAGAACG |  | Complem  entation of VdAHA1 |
| BIA1302-VdAHA1-RFP-R | CAGGAACAGGTGGTGGCGG ATTCACTAGTCAGGATCC | *Hind*Ⅲ | Complem  entation of VdAHA1 |
| VdAHA1-qPCR-F | GATCTGAGTTCTCCAAGCT |  | qRT-PCR |
| VdAHA1-qPCR-R | GGCGGCGCACGGGTAAAGGC |  | qRT-PCR |
| Vdβt-F | AACAACAGTCCGATGGATAATTC |  | qRT-PCR |
| Vdβt-R | GTACCGGGCTCGAGATCG |  | qRT-PCR |
| GhAct-F | CCTATGTTGCCCTGGACTATGAGC |  | qRT-PCR |
| GhAct-R | GGACAACGGAATCTCTCAGCTCC |  | qRT-PCR |
| VDAG_03486 | F:CCTGCCGTAGAGAAATGGAAGAC  R:GGTGGTGTAGCCTGGGAGAG |  | qRT-PCR |
| VDAG_10085 | F:GATTGGCTGATGGTTCCTTTTCC  R:ATGCTTCACTTATTTCACAGGTTCC |  | qRT-PCR |
| VDAG_03539 | F: TTGGTTGGGGCTTGACCT  R: GGAGGAGAACGCCCTTGAC |  | qRT-PCR |
| VDAG_03466 | F:GAATTGACAGTGTCTGATAGGCATGAC  R:GAATTGACAGTGTCTGATAGGCATGAC |  | qRT-PCR |
| VDAG_06939 | F:TGAGCGAGGACGAGGAGAAC  R:AGCAGCCGAACGACTGGTC |  | qRT-PCR |
| VDAG_05506 | F: CCGCTGTGTGTTTCTTTGAG  R: GCATAGGGTCGTTCGTTGA |  | qRT-PCR |
| VDAG_08194 | F: TCTATCTCCCATTCGGCATT  R:TGACTTTTCTTCTGGCTGCTT |  | qRT-PCR |
| VDAG_08181 | F: TCATCTTCGTCCTCGGCTA  R: CCCTGCTTCTTCTCATCCA |  | qRT-PCR |
| VDAG_05120 | F: TCCAAGCCGAGCACACTAT  R:CCTCACAACGGACAAAAGAAC |  | qRT-PCR |
| VDAG_08504 | F: CATTATCCAGCCCCTGATT  R: CCTTCTCCCGTCTCGTATT |  | qRT-PCR |
| VdNoxB | F:CAGCCCCCTTCGATCTCTCG  R:TCAGCTCTCTTTCCCCTGTC |  | qRT-PCR |
| VdPls1 | F:CAAATTGGCTGCCTACCTCA  R:CAAATTGGCTGCCTACCTCA |  | qRT-PCR |
| VdCrz1 | F:GAGAAGCCCTTCAGCACACTC  R:GAGAAGCCCTTCAGCACACTC |  | qRT-PCR |
| VdPlp | F: ATATCCGTCTGCTCGCCCTCG  R: GCCATAGGTCGGCTGCTGG |  | qRT-PCR |
| VdPf | F: CAGCCACTGCATCAACTAC  R: GAGGCGGCCTGTGAGGCC |  | qRT-PCR |
| VdEGR2 | F: CGACCTCGACCACCTCCACG  R: CCATGGCGCCGCCGGCGTTG |  | qRT-PCR |
| VdKin2 | F: TGTCCAGTCCCAGCACGATC  R: GGGATCGCGAAGACTCCTG |  | qRT-PCR |
| VdCf2 | F: CGAAGACCAGCGACGTCAACG  R: CGCTGGGCGGCGCTTTGGGC |  | qRT-PCR |
| hypothetical protein | F: CATTTGATGAGTATGAGGTTG  R: GTTGGTCCGCCCTTGATCTTGA |  | qRT-PCR |
| VaflM-qPCR-F | GACTGTCAATGCCATCGCC |  | qRT-PCR |
| VaflM -qPCR-R | CGGTGACCTTGATAACTT |  | qRT-PCR |
| VdSCD-qPCR-F | ATGCCCGCTTCCGAGTTC |  | qRT-PCR |
| VdSCD -qPCR-R | TTCCACACGCCGTCAATCTT |  | qRT-PCR |
| Vayg1-qPCR-F | GTTGCGACGAGTTCTTGT |  | qRT-PCR |
| Vayg1-qPCR-R | ACCATCACCTTGCCCATA |  | qRT-PCR |
| AD-VdHSP90-1 | F:CCATGGAGGCCAGTGAATTCatgtctgagactttcgagttccagg  R:AGCTCGAGCTCGATGGATCCttagtcgacctcctccatggc |  | Yeast |
| BD-VdAHA1 | F:TGGCCATGGAGGCCGAATTCatggtcctccataaccctaataactg  R:CGCTGCAGGTCGACGGATCCctacagaatggttccaaagccgaag |  | Yeast |
| nLUC-VdAHA1 | F:CGGGGGACGAGCTCGGTACCatggtcctccataaccctaataactg  R:GTCCCGGGGCGTCGAC ctacagaatggttccaaagccgaag |  | LCI |
| cLUC-Vd HSP90-1 | F:GTACGCGTCCCGGGGCGGTACCatgtctgagactttcgagttccagg  R:GAACGAAAGCTCTGCAGGTCGACttagtcgacctcctccatggc |  | LCI |

**Table S2**

| NCBI Gene Number | NCBI Gene description |
| --- | --- |
| VDAG_01302 | hypothetical protein |
| VDAG_01780 | ATP-dependent RNA helicase SUB2 |
| VDAG_06462 | hypothetical protein |
| VDAG_08282 | lactonohydrolase |
| VDAG_08343 | rhomboid family membrane protein |

**Table S3**

| NCBI Gene Number | NCBI Gene description |
| --- | --- |
| VDAG_04290 | hypothetical protein |
| VDAG_04645 | heat shock protein |
| VDAG_06465 | hypothetical protein |
| VDAG_08611 | hypothetical protein |

**Table S4**

| Name | Website |
| --- | --- |
| NCBI | https://www.ncbi.nlm.nih.gov |
| ENDscript/ESPript 3.0 | https://espript.ibcp.fr/ESPript/ESPript/index.php |

**Table S5**

| Name | NCBI Gene Number |
| --- | --- |
| VdAHA1 | VDAG_10067 |
| VdHSP90-1 | VDAG_ 04645 |
| VdNoxB | VDAG_09930 |
| VdPls1 | VDAG_01769 |
| VdCrz1 | VDAG_03208 |
| VdPlp | VDAG_00942 |
| VdPf | VDAG_08521 |
| VdEGR2 | VDAG_01363 |
| VdKin2 | VDAG_09024 |
| VdCf2 | VDAG_08721 |
| hypothetical protein | VDAG_07742 |
| VaflM | VDAG_00183 |
| VdSCD | VDAG_03393 |
| Vayg1 | VDAG_04954 |
